# Chronic kidney disease awareness: a cross-sectional study in primary care settings in Türkiye

**DOI:** 10.1007/s40620-025-02210-y

**Published:** 2025-03-08

**Authors:** Esra Gök, Mustafa Kürşat Şahin

**Affiliations:** Department of Family Medicine, School Medicine, Ondokuz Mayıs University, Samsun, Türkiye

**Keywords:** Chronic kidney disease, Chronic kidney insufficiency, Primary health care, Knowledge, Awareness

## Abstract

**Background:**

Raising awareness of chronic kidney disease (CKD) is essential for early detection and prevention, since the condition remains largely underdiagnosed, particularly in primary care settings. The present study aimed to evaluate awareness levels regarding the causes and symptoms of CKD among individuals receiving primary care.

**Methods:**

This cross-sectional study included 457 participants recruited from two primary care centers in Türkiye using systematic random sampling. The data were collected via face-to-face interviews using a pre-tested questionnaire between April and June 2023. Awareness levels were classified using Bloom’s cutoff points–high awareness (≥ 80%), moderate awareness (60–79%), and low awareness (< 60%).

**Results:**

The mean age of the participants was 42.3 ± 14.9 years. The study population consisted of 55.4% women, 51.2% of the participants were aged 18–39, 74.6% were married, and 53.4% held at least a university bachelor’s degree. As for CKD awareness, 64.1% were aware that CKD can be caused by pain medication, 56% that it can be caused by hypertension, and 48.8% that it can result from diabetes. Additionally, 58.6% were aware that swelling of the feet and ankles can represent a symptom of CKD. Awareness of the causes and symptoms of CKD was low in 78.6% of our participants (n = 359), moderate in 17.5% (*n* = 80) and high in 3.9% (*n* = 18). Individuals who recalled having been informed about these causes and symptoms by their primary care physicians exhibited higher awareness. No significant differences in awareness were observed across different sociodemographic groups. A positive correlation was observed between awareness of the causes of CKD and awareness of its symptoms.

**Conclusions:**

Awareness of CKD causes and symptoms among the participants was limited, nearly half being unaware of key risk factors such as painkiller use, obesity, hypertension, smoking, diabetes, and herbal product use. Increased education, particularly by primary care physicians, may improve awareness and early detection rates.

**Graphical abstract:**

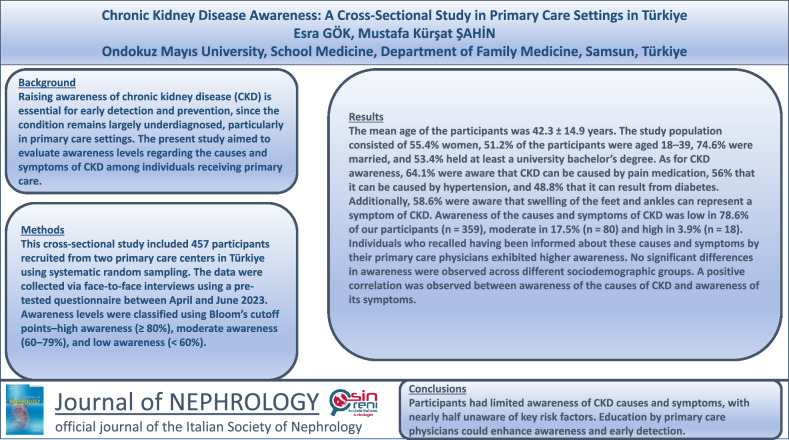

## Introduction

Chronic kidney disease (CKD) is becoming increasingly recognized as a significant global public health problem. The prevalence is rising due to factors such as aging populations and increasing rates of diabetes, hypertension, and obesity. Approximately 850 million people worldwide are estimated to suffer from CKD, a figure that is expected to rise still further, especially in low- and middle-income countries. The morbidity and mortality associated with CKD are also alarming, the disease representing the third fastest-growing cause of death worldwide. It is projected to become the fifth leading cause of years of life lost worldwide by 2040 [1]. The World Kidney Day campaign in 2020 highlighted the importance of preventive interventions for CKD. Progression can be prevented and delayed with lifestyle changes, early treatment, and access to basic diagnostics [2].

The global increase in CKD is primarily driven by the growing prevalence of diabetes, hypertension, and obesity, and by the aging of the populations. However, in some regions, other causes such as infections and the use of herbal remedies, as well as environmental toxins, are important [3]. Despite its serious health impacts, awareness of CKD remains critically low, particularly among high-risk groups. This lack of awareness frequently results in delayed diagnosis, increased morbidity, and a significant rise in healthcare costs [1, 4]. Studies have shown that up to 90% of individuals with CKD are unaware of their condition, which delays treatment and worsens disease progression. CKD awareness levels in Türkiye are similarly low. A study conducted by the Turkish Nephrology Association in 2009 across 23 Turkish provinces reported a CKD awareness level of 1.6%. However, according to the “Kidney Health Bus Project” carried out in 21 provinces in 2010, public awareness of CKD had risen to 5.7% [5]. The persistently low awareness of CKD in Türkiye, despite initiatives like the “Kidney Health Bus Project,” highlights the need for further research on this public health issue. Self-awareness of CKD is notably low among patients, only 8% being aware of their condition despite having access to publicly funded healthcare [6–8]. Studies from Ethiopia have revealed that only 12.9% of patients with diabetes and hypertension were aware of CKD risks and symptoms [9]. Public awareness of CKD in Iran was even lower, with fewer than 15% of participants knowing about the disease and the main risk factors [10]. A study from Germany likewise reported high unawareness, with over 75% of the population being unaware of their CKD status [11]. In low- and middle-income countries, the estimated awareness is approximately 6% in the general population and 10% in high-risk cohorts, comparable to figures seen in high income countries [12]. For instance, data from the Kidney Early Evaluation Program screening programs in the USA showed that only 7% to 9% of participants were aware of their CKD status [13].

A condition frequently overshadowed by better-known health risks, CKD requires a multifaceted approach, not only for prevention but also for careful management throughout its various stages. Raising awareness at the primary care level has the potential to significantly affect secondary and tertiary prevention strategies, which focus on halting disease progression and managing long-term complications. As CKD continues to affect millions of lives, efforts to amplify public understanding of its causes and symptoms are not only a public health necessity, but also a moral imperative. Primary care physicians, acting as gatekeepers for early detection, play a crucial role in providing patients with the knowledge that may prevent this silent disease from becoming an irreversible condition [2].

This study was intended to evaluate the awareness levels of individuals presenting to primary care institutions concerning the causes and symptoms of CKD. Unlike earlier nationwide efforts, this research provides localized data and investigates awareness levels in a specific population, including young and educated individuals who represent a critical demographic for shaping future health behaviors. This approach addresses a significant gap in the literature by emphasizing primary care settings as a strategic context for enhancing CKD awareness. Understanding these awareness levels is crucially important for developing effective public health strategies to enhance early detection, improve management, and ultimately reduce the societal burden of CKD. We expect that the findings of this study will contribute to the formulation of targeted interventions that enhance CKD awareness and promote timely diagnosis and treatment.

## Materials and methods

### Study location and duration

This cross-sectional study was conducted at the Pelitköy Education Family Health Center and the Aziziye Education Family Health Center, both affiliated with the Department of Family Medicine at the Ondokuz Mayıs University Faculty of Medicine. The primary outcome was to assess awareness of the causes and symptoms of CKD in primary care settings in Türkiye.

### Study population and sample size

The study population consisted of individuals aged 18–65 years registered at the Pelitköy and Aziziye Family Health Centers. The study was conducted in two family health centers located near a university campus. These centers predominantly serve university students, employees, and their families, which may affect the generalizability of the results to other primary care settings. The participants were required to fall within this age range and be willing to participate in the study. Healthcare workers and individuals with barriers to communication, such as language difficulties, cognitive impairments, or sensory impairments related to hearing or vision, along with those over 65 or reliant on caregivers, were excluded from the study. At the commencement of the study, 5296 individuals aged 18–65 were registered at the two-unit Pelitköy Family Health Center, while 2162 individuals from the same age group were registered at the two-unit Aziziye Education Family Health Center. The populations served by the two clinics were similar in characteristics. The sample size was calculated using OpenEpi software, applying finite population correction for a total population of 7458. Reported CKD awareness rates in previous studies have ranged from 2 to 25%, and these values were considered when determining the sample size [5–13]. The hypothesized prevalence of the outcome (CKD awareness) was set at 10%, with a margin of error of ± 3%. A 95% confidence level was used for the calculation, yielding a required sample size of 366 participants.

The Turkish healthcare system is built around a universal coverage model, in which primary care services are readily accessible to all citizens through a network of family health centers which serve as the first point of contact for individuals seeking medical care. Patients with known risk factors for CKD, such as hypertension, diabetes, or obesity, attend regular check-ups with primary care physicians, who play a critical role in managing these conditions through regular monitoring and preventive measures. Although individuals with such risk factors constitute key populations in CKD prevention, this study did not specifically target these high-risk groups.

### Data collection tools

The data collection instrument was a questionnaire specifically designed for this study by the research team. This was developed following a comprehensive review of previous studies evaluating awareness of the causes and symptoms of CKD and associated factors [14–16]. During the development process, feedback was obtained from two nephrology specialists, two family medicine specialists, and a linguist. Additionally, a validation test was performed on the revised version of the questionnaire.

The draft questionnaire was content-validated by considering the length, clarity, language, timeliness, bias, and appropriateness of the questions. This validation involved consultations with two nephrology and three family medicine specialists. Feedback was also collected from six members of the general public, consisting of healthy adults of both sexes, and six individuals with no medical histories. These participants were invited to provide input concerning the clarity, relevance, appropriateness, length, and time required to complete the questionnaire. In order ensure the reliability of the questionnaire, all suggested modifications were carefully considered by the research team. A pilot test was subsequently conducted using the final version of the questionnaire, involving 15 participants who were asked to identify any unclear questions or statements. Following this process, the finalized questionnaire consisted of three sections. The final form, prepared in Turkish, is included as an attachment.

Participants were selected based on their willingness to participate, indicated by verbal consent, and their order of arrival at the outpatient clinic. The questionnaire was administered in a face-to-face setting. The initial section included 11 items concerning sociodemographic data, such as age, gender, marital status, and educational background. The second section consisted of 20 questions assessing awareness of the causes of CKD, while the final section contained 20 items evaluating awareness of CKD symptoms. All items were evaluated using a three-point Likert scale. The options were 1-Disagree, 2-Neutral, and 3-Agree. The “Disagree” option was regarded as an incorrect answer, the “Neutral” response was categorized as incorrect in order to emphasize the importance of definitive awareness of CKD causes and symptoms, while the “Agree” option was considered correct. Incorrect answers were scored zero and correct answers were scored one. Awareness levels were categorized according to Bloom’s cutoff points. Scores of 80% and above were classified as high awareness, scores of 60%-79% as moderate awareness, and scores below 60% as low awareness [17].

### Data collection procedure

The participants were not approached through their primary care physicians. Instead, we included individuals who presented to the Pelitköy Education Family Health Center and the Aziziye Education Family Health Center during the study period, from April to June 2023. It is important to note that the study focused solely on adults aged 18 and older, with recruitment occurring on-site. Participants were recruited sequentially in order of presentation. Once verbal consent had been obtained, the questionnaire was administered face-to-face by trained interviewers. In order to minimize interviewer bias, all the interviewers were trained and provided with standardized instructions for administering the survey.

### Statistical analysis

The data were analyzed on IBM SPSS version 21.0 software. Descriptive statistics, including frequencies, percentages, means, and standard deviations, were calculated. Chi-square tests were used to compare categorical variables, and correlations were assessed via correlation analysis.

### Ethical considerations

Ethical approval was obtained from the Clinical Research Ethics Committee of Ondokuz Mayıs University (approval no. OMU KAEK 2023/76). Participant confidentiality was maintained by anonymizing the data and securing all electronic records. Informed consent procedures were followed, with verbal consent being documented by the interviewers.

## Results

Four hundred fifty-seven individuals with a mean age of 42.3 ± 14.9 years participated in this study. Women constituted 55.4% (*n* = 253) of the participants, 51.2% (*n* = 234) were aged 18–39, 74.6% (*n* = 341) were married, and 53.4% (*n* = 244) had been educated to university level or higher. In terms of income status, 51.9% (*n* = 237) reported having an income equal to expenses. Additionally, 30.4% (*n* = 139) were smokers, 11.4% (*n* = 52) consumed alcohol, and 12.5% (*n* = 57) had been informed by their family physician about the causes and symptoms of CKD (Table 1).

**Table 1:** Participant characteristics

Variable	Category	*n* (%)
Sex	Female	253 (55.4)
	Male	204 (44.6)
Age (years)	18–39	234 (51.2)
	40–59	149 (32.6)
	60–65	74 (16.2)
Marital status	Married	341 (74.6)
	Not married	116 (25.4)
Education status	Literate (but no formal schooling)	6 (1.3)
	Primary school graduate	57 (12.5)
	Middle school graduate	27 (5.9)
	High school graduate	123 (26.9)
	University graduate	244 (53.4)
Income status	Income less than expenditure	116 (25.4)
	Income equal to expenditure	237 (51.9)
	Income exceeding expenditure	104 (22.8)
Smoking status	Smoker	139 (30.4)
	Non-smoker	318 (69.6)
Alcohol use	Yes	52 (11.4)
	No	405 (88.6)
Having been informed by a primary care physician about the causes and symptoms of chronic kidney diseases	Yes	57 (12.5)
	No	400 (87.5)

The most frequently correctly identified cause of CKD was a history of acute kidney disease (68.7%). Additionally, 56% of the participants were aware that high blood pressure can cause CKD, and 48.8% that diabetes can do so (Table 2). The most frequently identified symptoms of CKD were frequent urination at night (66.7%), swelling in the feet and ankles (58.6%), and uncontrollable high blood pressure (51.9%). In addition, 50.1% of the participants were aware of fatigue as a symptom, 35.4% of nausea and vomiting, and 35% of loss of appetite (Table 3).

**Table 2:** Percentage of correct responses to statements regarding awareness of the causes of chronic kidney disease

Chronic kidney disease causes awareness statements	*n* (%)
Anemia can cause chronic kidney disease	113 (24.7)
Contrast agents used in medical imaging (CT and MRI) can cause chronic kidney disease	148 (32.4)
Infections such as hepatitis and HIV (AIDS) can cause chronic kidney disease	161 (35.2)
Heart diseases can cause chronic kidney disease	194 (42.5)
Herbal products can cause chronic kidney disease	201 (44.0)
Recurrent kidney stones can cause chronic kidney disease	202 (44.2)
Advanced age can cause chronic kidney disease	210 (46.0)
Drinking alcohol can cause chronic kidney disease	216 (47.3)
Diabetes can cause chronic kidney disease	223 (48.8)
Smoking can cause chronic kidney disease	225 (49.2)
Cystic diseases of the kidney can cause chronic kidney disease	247 (54.0)
Recurrent kidney and urinary tract infections can cause chronic kidney disease	254 (55.6)
High blood pressure can cause chronic kidney disease	256 (56.0)
Obesity can cause chronic kidney disease	264 (57.8)
Inflammation of the thin urinary ducts inside the kidney can cause chronic kidney disease	279 (61.1)
Diseases that damage the filtering units of the kidney can cause chronic kidney disease	289 (63.2)
Low water consumption can cause chronic kidney disease	290 (63.5)
The use of painkillers can cause chronic kidney disease	293 (64.1)
Hereditary kidney disease in the family can cause chronic kidney disease	307 (67.2)
Acute kidney failure can cause chronic kidney disease	314 (68.7)

*CT* computed tomography, *MRI* magnetic resonance imaging, *HIV (AIDS)* human immunodeficiency virus (acquired immune deficiency syndrome)

**Table 3:** Percentage of correct responses to statements regarding awareness in the symptoms of chronic kidney disease

Chronic kidney disease symptoms awareness statements	*n* (%)
Paralysis can be a symptom of chronic kidney disease	72 (15.8)
Decreased sexual desire or infertility can be a symptom of chronic kidney disease	92 (20.1)
Difficulty focusing (concentration) can be a symptom of chronic kidney disease	96 (21.0)
Muscle cramps can be a symptom of chronic kidney disease	112 (24.5)
Numbness and tingling in the hands and feet may be a symptom of chronic kidney disease	116 (25.4)
Dry and itchy skin may be a symptom of chronic kidney disease	117 (25.6)
Anemia may be a symptom of chronic kidney disease	120 (26.3)
Increased levels of potassium in the blood can be a symptom of chronic kidney disease	123 (26.9)
Heart disease (heart failure, arrhythmia, etc.) can be a symptom of chronic kidney disease	132 (28.9)
Weak bones and an increased risk of bone fractures can be a sign of chronic kidney disease	133 (29.1)
Chest pain can be a symptom of chronic kidney disease if fluid builds up around the pericardium	138 (30.2)
Shortness of breath can be a symptom of chronic kidney disease if fluid builds up in the lungs	146 (31.9)
Sleep disturbance may be a symptom of chronic kidney disease	151 (33.0)
Swelling around the eyes may be a sign of chronic kidney disease	152 (33.3)
Loss of appetite can be a symptom of chronic kidney disease	160 (35.0)
Nausea and vomiting can be a symptom of chronic kidney disease	162 (35.4)
Fatigue can be a symptom of chronic kidney disease	229 (50.1)
High blood pressure that is difficult to control can be a sign of chronic kidney disease	237 (51.9)
Swelling of the feet and ankles can be a sign of chronic kidney disease	268 (58.6)
Frequent urination at night may be a symptom of chronic kidney failure	305 (66.7)

There were no significant differences in awareness levels of causes or symptoms of CKD in terms of sex, marital status, education level, income status, smoking status, or alcohol consumption (*p* > 0.05). However, participants aged 18–39 years had significantly lower CKD cause awareness levels compare to other age groups (*p* < 0.033). Participants who recalled being informed by their primary care physicians about the causes and symptoms of CKD exhibited significantly higher levels of awareness regarding CKD symptoms (*p* < 0.006). Awareness of the causes of CKD was low in 60.2% of the participants (*n* = 275), moderate in 26.7% (*n* = 122), and high in 13.1% (*n* = 60). Awareness of the symptoms of CKD was low in 85.8% (*n* = 392), moderate in 11.1% (*n* = 51), and high in 3.1% (*n* = 14) (Table 4). Awareness of the causes and symptoms of CKD was low in 78.6% of our participants (*n* = 359), moderate in 17.5% (*n* = 80) and high in 3.9% (*n* = 18).

**Table 4:** Level of awareness of causes and symptoms of chronic kidney diseases according to various participant characteristics

Variables	Category	Level of awareness of causes of CKD			p	Level of awareness of symptoms of CKD			p
		Low	Moderate	High		Low	Moderate	High	
		n (%) 275 (60.2)	n (%) 122 (26.7)	n (%) 60 (13.1)		n (%) 392 (85.8)	n (%) 51 (11.1)	n (%) 14 (3.1)	
Sex	Female	156 (61.7)	67 (26.5)	30 (11.9)	0.633	217 (85.8)	30 (11.9)	6 (2.4)	0.567
	Male	119 (58.3)	55 (27.0)	30 (14.7)		175 (85.8)	21 (10.3)	8 (3.9)	
Age (years)	18–39	156 (66.7)	48 (20.5)	30 (12.8)	**0.033**	201 (85.9)	27 (11.5)	6 (2.6)	0.65
	40–59	81 (54.4)	48 (32.2)	20 (13.4)		131 (87.9)	13 (8.7)	5 (3.4)	
	≥ 60	38 (51.4)	26 (35.1)	10 (13.5)		60 (81.1)	11 (14.9)	3 (4.1)	
Marital status	Married	199 (58.4)	99 (29.0)	43 (12.6)	0.153	294 (86.2)	36 (10.6)	11 (3.2)	0.747
	Not married	76 (65.5)	23 (19.8)	17 (14.7)		98 (84.5)	15 (12.9)	3 (2.6)	
Education status	High school and below	135 (63.4)	57 (26.8)	21 (9.9)	0.14	186 (87.3)	19 (8.9)	8 (3.8)	0.282
	University and above	140 (57.4)	65 (26.6)	39 (16.0)		206 (84.4)	32 (13.1)	6 (2.5)	
Income Status	Income less than expenditure	76 (65.5)	26 (22.4)	14 (12.1)	0.168	99 (85.3)	13 (11.2)	4 (3.4)	0.986
	Income equal to expenditure	144 (60.8)	67 (28.3)	26 (11.0)		205 (86.5)	25 (10.5)	7 (3.0)	
	Income exceeding expenditure	55 (52.9)	29 (27.9)	20 (19.2)		88 (84.6)	13 (12.5)	3 (2.9)	
Smoking status	Smoker	83 (59.7)	36 (25.9)	20 (14.4)	0.862	123 (88.5)	12 (8.6)	4 (2.9)	0.513
	Non-smoker	192 (60.4)	86 (27.0)	40 (12.6)		269 (84.6)	39 (12.3)	10 (3.1)	
Alcohol use	Yes	34 (65.4)	10 (19.2)	8 (15.4)	0.424	48 (92.3)	3 (5.8)	1 (1.9)	0.355
	No	241 (59.5)	112 (27.7)	52 (12.8)		344 (84.9)	48 (11.9)	13 (3.2)	
Having been informed by a primary care physician about the causes and symptoms of chronic kidney diseases	Yes	36 (63.2)	11 (19.3)	10 (17.5)	0.301	50 (87.7)	2 (3.5)	5 (8.8)	**0.006**
	No	239 (59.8)	111 (27.8)	50 (12.5)		342 (85.5)	49 (12.3)	9 (2.3)	

A significant association was observed between levels of awareness of the causes of CKD and levels of awareness of symptoms (*p* < 0.001). Participants with low awareness of causes also exhibited low awareness of symptoms. Awareness of CKD symptoms improves in line with awareness of causes, indicating a positive correlation between cause and symptom awareness.

## Discussion

Chronic kidney disease prevalence is increasing worldwide, yet public awareness remains limited [4]. CKD can be prevented or diagnosed early through the implementation of preventive measures, thereby increasing quality of life and reducing healthcare costs. Primary care physicians play crucial roles in educating and raising awareness among the public, as well as in implementing preventive approaches [18]. This study assessed the awareness of individuals presenting to primary care regarding the causes and symptoms of CKD. The majority of participants exhibited low awareness of both the causes and symptoms of CKD. The great majority of participants were unaware of either the causes or symptoms of CKD. Similar results have also been obtained in previous studies [5–8, 12, 13].

The most widely recognized risk factors for CKD include the use of pain relievers and low water consumption. Previous studies have also shown that participants are less aware of these risk factors [10, 14, 19]. Recent studies emphasizing the importance of water consumption, both nationally and globally, along with widespread warnings by physicians regarding the deleterious outcomes of excessive painkiller use, may have contributed to this finding.

The participants in this study exhibited low awareness of the potential role of obesity, hypertension, smoking, diabetes, advanced age, and the use of herbal products. These findings are closely aligned with global public health concerns regarding obesity and its complications, highlighting a major need for enhanced public education [20]. Some studies involving participants with hypertension or at risk for CKD have reported increasing awareness among these groups regarding the effects of hypertension [14, 16, 21, 22]. However, others have reported lower awareness levels concerning the role of hypertension in the etiology of CKD [15, 23–25]. Since hypertension is one of the major causes of CKD, low awareness of the complications of this chronic condition may affect a significant proportion of the population. Providing patients with information about salt consumption, medication adherence, and potential complications of hypertension may enhance their awareness and facilitate cooperation in the management of chronic diseases. Fewer than half of the participants in the current study were aware that smoking can cause CKD, a finding consistent with the previous literature [14, 16, 21, 26]. Increasing global awareness campaigns about smoking and its adverse effects may be effective in reducing healthcare costs and improving individuals’ quality of life. Fewer than half of our participants were aware that diabetes can cause CKD. This finding is also consistent with the literature [9, 16, 21, 23]. Considering the increasing prevalence of diabetes and the silent progression of its complications, primary care physicians have a duty to regulate blood sugar levels and regularly monitor individuals diagnosed with the condition, as well as to raise awareness among the patients registered with them about its complications. The majority of our participants were unaware of the renal effects of advanced age. In contrast, other studies have shown that a greater proportion of their participants were aware that advanced age can cause CKD [14, 16, 26]. This may be attributable to the majority of the participants in this study being in the younger age group and possessing lower awareness of the effects of aging. More than half of our participants were also not aware that herbal product use may cause CKD. In contrast, Assiry et al. reported a higher rate of awareness [14]. This may be due to the use of herbal products and supplements having increased in recent years both in Türkiye and worldwide, although the general public is unaware of their potential side-effects. In Oluyombo et al.’s study, a smaller proportion of participants were aware of this risk factor [19]. These discrepancies may reflect varying social and cultural attitudes toward herbal products across different regions.

The most widely recognized CKD symptom in this study was swelling in the feet and ankles, which was correctly identified by more than half of the participants. Similar findings were reported in other studies [14, 16, 26, 27]. The widespread awareness of the role of the kidney in fluid regulation probably contributed to this level of knowledge. Half the participants recognized fatigue as a potential CKD symptom; in this regard Assiry et al. reported lower awareness [14] and Alobaidi et al. and Gheewala et al. higher awareness [15, 27]. This variation may be due to the non-specific nature of fatigue, which is common to many conditions.

Analysis of awareness levels of CKD causes and symptoms according to sociodemographic characteristics revealed no gender difference. Other studies have also reported no gender differences [14, 22, 27, 28]. This lack of difference between women and men may be due to both enjoying equal access to health education. At difference, the studies by Oluyombo et al. and Ng et al. reported increased CKD awareness among male participants [19, 29]. This discrepancy may be attributed to differences in access to education between women and men across countries.

Examination of awareness levels of CKD causes according to sociodemographic characteristics showed that awareness was lower in participants aged 18–39 than in those from other age groups. This may be because participants in this age group feel healthy and do not perceive the need to monitor themselves for chronic disease symptoms. Additionally, health concerns among elderly individuals may have contributed to their increased awareness of disease prevention. In contrast, some studies have reported no significant differences in awareness across age groups, suggesting that more research is needed to understand these variations [15, 21, 22, 27, 28]. Other studies have reported increased CKD awareness in younger age groups [19, 29]. This may be due to the fact that studies showing higher awareness in younger age groups were conducted earlier, and that awareness among the elderly population has increased over time.

No differences were found in levels of awareness of CKD causes and symptoms in term of participants’ education levels, marital status, or income levels. This finding is also consistent with the literature [22, 28]. This suggests that, irrespective of these sociodemographic characteristics, the population in general possesses low awareness of CKD and requires education on the subject.

When levels of awareness of CKD symptoms were analyzed according to sociodemographic characteristics, participants who recalled being informed of them by a primary care physician exhibited higher awareness levels of those symptoms. This highlights the importance of primary care physicians in increasing patient awareness. Yao et al. highlighted the importance of, and need for, short videos to raise public awareness of CKD [30]. Jennette et al. mentioned the need for educational measures, including media campaigns, to increase public awareness of CKD and raise awareness. They also emphasized that poor communication between health professionals and patients should be improved [25].

The participants in this study demonstrated a notable lack of awareness of the underlying causes of CKD, including factors such as obesity, hypertension, smoking, diabetes, advanced age, and the use of herbal products. Furthermore, the majority of the participants exhibited low awareness of the causes and symptoms of CKD. In order to increase awareness of CKD in the primary care setting, physicians should inform the patients registered with them about the risks associated with obesity, encourage obese individuals to lose weight, educate hypertensive and diabetic patients about the complications of their conditions, monitor medication compliance, motivate smokers to quit, and caution against the uncontrolled and excessive use of herbal products. Additionally, regular follow-up procedures for individuals at risk of developing CKD will facilitate early diagnosis of the disease.

The findings of this study suggest a pervasive lack of CKD awareness among individuals presenting to primary care centers in Türkiye, particularly in terms of recognizing key risk factors and symptoms. These findings align with global trends in CKD awareness, particularly in regions where primary care plays a key role in preventive healthcare, such as in Southern Europe and parts of the Middle East [14, 19, 23, 29]. These findings indicate that despite different sociodemographic and sociocultural characteristics, the community as a whole possesses low awareness of CKD.

A significant association was observed between the participants’ levels of awareness of CKD causes and symptoms, those with higher levels of awareness of CKD causes also exhibiting higher awareness levels of symptoms. Assiry et al. also reported a positive correlation between awareness of CKD causes and symptoms [14]. This finding shows that individuals with a high level of awareness of the causes of CKD also have a high level of awareness of its symptoms. It may also be inferred that awareness of the causes of the disease enhances awareness of the symptoms.

The findings of this study must be considered in light of its limitations. First, it should be noted that the data were collected from a limited geographic area, which may not be fully representative of all primary care settings in Türkiye. This geographic limitation may affect the generalizability of the findings to other regions with disparate population characteristics and healthcare systems. Another key limitation of this study is the lack of detailed information regarding the study setting. Although we acknowledge that generalizability may be limited, it would have been desirable to specify whether the population was predominantly urban, semi-urban, or rural. Future research should aim to provide a clearer description of the study environment in order to enhance the relevance and potential impact of the findings. Furthermore, the research may have been subject to response bias, as individuals with higher levels of education and greater health awareness were potentially more likely to participate. This may have resulted in an overestimation of CKD awareness among the general population. Although the questionnaire used in this study was pre-tested to ensure clarity and relevance, the tools employed were not formally validated. In addition, the education level of the participants may have been higher than that of the general population, as the educational family health center where we collected the study data was located near a university. Non-validated instruments can introduce bias, potentially affecting the accuracy of the data collected. In order to address this limitation in future research, studies should endeavor to include a more diverse sample of participants, encompassing a broader range of geographic locations and sociodemographic characteristics. Another limitation of the study is its reliance on self-reported data, which may be subject to inaccuracies due to recall bias or social desirability bias. It is possible that participants may have over-reported their awareness of CKD risk factors and symptoms, particularly if they perceived such awareness as being socially desirable. Future studies could address this issue by incorporating objective measures of CKD awareness, such as quizzes or awareness tests administered in a controlled setting. Finally, the study did not assess the effectiveness of different educational interventions on improving CKD awareness. Understanding which strategies are most effective in raising awareness and promoting early detection would be valuable to the development of future public health initiatives. Future research should consider conducting intervention studies that evaluate the impact of various educational approaches on CKD awareness and outcomes.

## Conclusion

The majority of participants in this study exhibited a low level of awareness of the causes and symptoms of CKD. Most participants correctly answered fewer than half the questions related to the causes and symptoms. Awareness of CKD symptoms increased in line with awareness of the causes.

Nearly half the participants in this research were unaware that the use of painkillers, as well as the presence of obesity and hypertension may represent causes of CKD, and over half were unaware that smoking, diabetes, and the use of herbal products may lead to the condition. Similarly, almost half were unaware that swelling in the feet and ankles and uncontrolled high blood pressure may constitute symptoms of CKD, and over half were unaware that puffiness around the eyes may likewise be a symptom. This lack of awareness regarding common causes of CKD such as diabetes and hypertension underscores the importance of raising public awareness to ensure early diagnosis and prevention of CKD.

Primary care physicians should also inform the general population about the symptoms of CKD, ensuring that awareness is raised across all individuals, irrespective of specific risk factors. The symptoms are non-specific, and further tests and examinations should be conducted for the early diagnosis of CKD when required. Patients should also be referred to a higher level of care if needed. Future interventions might usefully focus on brief but high-impact educational materials delivered in primary care settings, such as posters, brochures, and short informational videos in order to effectively increase CKD awareness. Furthermore, as with any health topic, improving communication between health professionals and patients can increase the quality of health services, ensure regular check-ups and follow-ups, and provide opportunities to increase patient awareness.

A simple question such as “Did you know that diabetes, hypertension, or obesity can lead to chronic kidney disease?” may effectively raise patient awareness concerning the condition. Increased awareness can improve adherence to medications, dietary guidelines, and lifestyle changes, helping patients manage their existing conditions more effectively. This approach can also help prevent complications and reduce overall healthcare costs.

## Data Availability

The datasets generated and/or analyzed during the current study are available from the corresponding author upon reasonable request.
